# Aberrant splicing events caused by insertion of genes of interest into expression vectors

**DOI:** 10.7150/ijbs.72408

**Published:** 2022-07-18

**Authors:** Yun Cheng, Xiao-Zhuo Kang, Pearl Chan, Zi-Wei Ye, Chi-Ping Chan, Dong-Yan Jin

**Affiliations:** 1 School of Biomedical Sciences, The University of Hong Kong, 21 Sassoon Road, Pokfulam, Hong Kong; 2 State Key Laboratory of Liver Research, The University of Hong Kong, 21 Sassoon Road, Pokfulam, Hong Kong; 3 Department of Microbiology, The University of Hong Kong, 102 Pokfulam Road, Pokfulam, Hong Kong

**Keywords:** Aberrant RNA splicing, lentiviral vector, gene therapy, exon-exon junction, splice donor site, V5 tag

## Abstract

**Background:** Expression of genes of interest from plasmids or lentiviral vectors is one of the most common tools in molecular and gene therapy. Aberrant splicing between the inserted gene of interest and downstream vector sequence has not been systematically analyzed.

**Methods:** Formation of aberrant fusion transcripts and proteins was detected by RT-PCR, sequencing, Western blotting and mass spectrometry. Bioinformatic analysis was performed to identify all human and mouse genes prone to vector-dependent aberrant splicing. Selected genes were experimentally validated.

**Results:** When we expressed human *FACI* in cultured cells, an aberrant splicing event was found to occur between *FACI* transcript and downstream plasmid sequence through one exon-exon junction in *FACI* that accidentally contributes a splice donor site. To explore whether this could be a general phenomenon, we searched the whole human and mouse genomes for protein-coding genes that harbor an exon-exon junction resembling a splice donor site. Almost all genes prone to this type of aberrant splicing were identified. A total of 17 genes among the hits were randomly selected for experimental validation. RT-PCR and sequencing results verified that 13 genes were aberrantly spliced on the identified exon-exon junctions. In addition, all 17 genes were aberrantly spliced on their V5 tag sequence. Aberrant fusion protein expression from all 17 genes was validated by immunoblotting. Aberrant splicing was prevented by recoding the V5 tag or the splice sites.

**Conclusions:** Our study revealed an unexpectedly high frequency of vector-dependent aberrant splicing events. Aberrant formation of the resulting fusion proteins could undermine the accuracy of gain-of-function studies and might cause potential side effects when the therapeutic gene is expressed *in vivo*. Our work has implications in improving vector construction and epitope tagging for gene expression and therapy.

## Introduction

RNA splicing is a process that transforms the precursor messenger RNA (pre-mRNA) into mature ready-to-translate mRNA. During splicing, introns in pre-mRNA are removed and exons, no matter coding or non-coding, are joined together to form the final mRNA. RNA splicing is a highly conserved strategy that exists in all forms of life, but with varied extents and processes 1. In eukaryotes, spliceosomes, a large ribonucleoprotein complex, mediate RNA splicing. Spliceosomes recognize specific donor and acceptor sequences of introns in pre-mRNA, and catalyze the removal of introns and joints of exons 2. In humans, about 99% splice recognition sites are the “GU-AG” type with GU dinucleotide at the 5′ splice donor site and AG at the 3′ acceptor site 3. The consensus sequence of this type is “AN|GUpuAG” where N = A, U, C or G; pu = A or G; and “|” indicates the cleavage site 4. A minority of splicing occurs at other non-canonical recognition sites, such as “GC-AG” (~0.56% in humans) and “AU-AC” (~0.09%) 5. In addition, there are numerous cryptic splice sites, also known as pseudo-splice sites, on the genome. The sequence of a cryptic splice site resembles a splice recognition motif. However, due to the lack of the cis-regulatory elements for splicing, cryptic splice sites cannot function in normal conditions 6. RNA splicing plays an important role in governing protein expression. A single mutation on the splice recognition sites could interrupt the splicing process and lead to aberrant protein production 7. It is estimated that 15-50% of disease-causing mutations in human monogenetic diseases result in abnormal RNA splicing 8.

Expression of foreign genes of interest (GOI) in target cells is not only a basic tool for studying gene function but also the foundation of transgenic gene therapy 9, 10. Several strategies have been developed to express foreign genes in cultured cells or *in vivo*. Whereas rapid and high-yield protein production can be achieved via transient transfection, construction of stable cell lines, in which the foreign GOI has been integrated into cellular genome, provides a more reliable approach for long-term protein expression 11. Lentiviral or retroviral gene transduction is a common and highly efficient method for stable expression of foreign genes in host cells via gene integration 12. To achieve stable expression and to remove non-expressing cells, selective markers are commonly exploited 13.

SDS-PAGE followed by immunoblot analysis is the most convenient method to verify successful expression of foreign genes. However, it is not uncommon that the actual size of a target protein in immunoblots does not match the calculated molecular mass. Several reasons could lead to such discrepancy 14. First, the large-sized protein complex or polymer may still be present in the protein samples, because protein-protein interactions are not completely disrupted. Incomplete denaturation during protein lysis could result in only partial loss of the noncovalent interaction of proteins. Likewise, incomplete reduction, due to insufficient reductant (such as β-mercaptoethanol or dithiothreitol) in lysis buffer, could lead to incomplete disruption of intermolecular disulfide bonds. Second, post-translational modifications (PTM) of proteins, such as ubiquitination, sumoylation, phosphorylation and glycosylation may also alter migration of protein bands. Third, the protein could undergo degradation or cleavage, which generates smaller-sized protein species. In addition, net charges of proteins that are not eliminated in the lysis process could also contribute to the mobility shift in SDS-PAGE 15, 16.

FACI, which stands for fasting- and CREB-H-induced protein and is also known as C11ORF86, is a novel enterocyte- and hepatocyte-enriched protein that suppresses lipid absorption 17. When we characterized FACI for function, both human FACI (hFACI) and mouse FACI (mFACI) were transiently or stably expressed in target cell lines. Appearance of one extra protein band unique to hFACI in immunoblots prompted us to further investigate its origin. The band of greater size can only be explained by aberrant splicing between *FACI* open reading frame (ORF) and the downstream plasmid sequence. The exon-exon junction (EJ) sequence “AG|GUAAG” in *FACI* coincidently fits the splicing donor site “AN|GUpuAG” 18, which mediates the aberrant splicing of *FACI* transcript. We went on to perform a whole-genome analysis and identified all genes with splice donor-like EJs. Aberrant splicing was validated experimentally for a randomly selected group of these genes, and it occurs more frequently than expected during transient and stable expression of target genes. Normal functions of the proteins of interest might be altered or lost in the fusion proteins resulting from aberrant splicing. Aberrant splicing might also account for unexpected protein band shift in immunoblot analysis.

## Results

### Appearance of an unusual migration band in SDS-PAGE analysis of recombinant human FACI expressed from cultured cells

**Figure 1:**
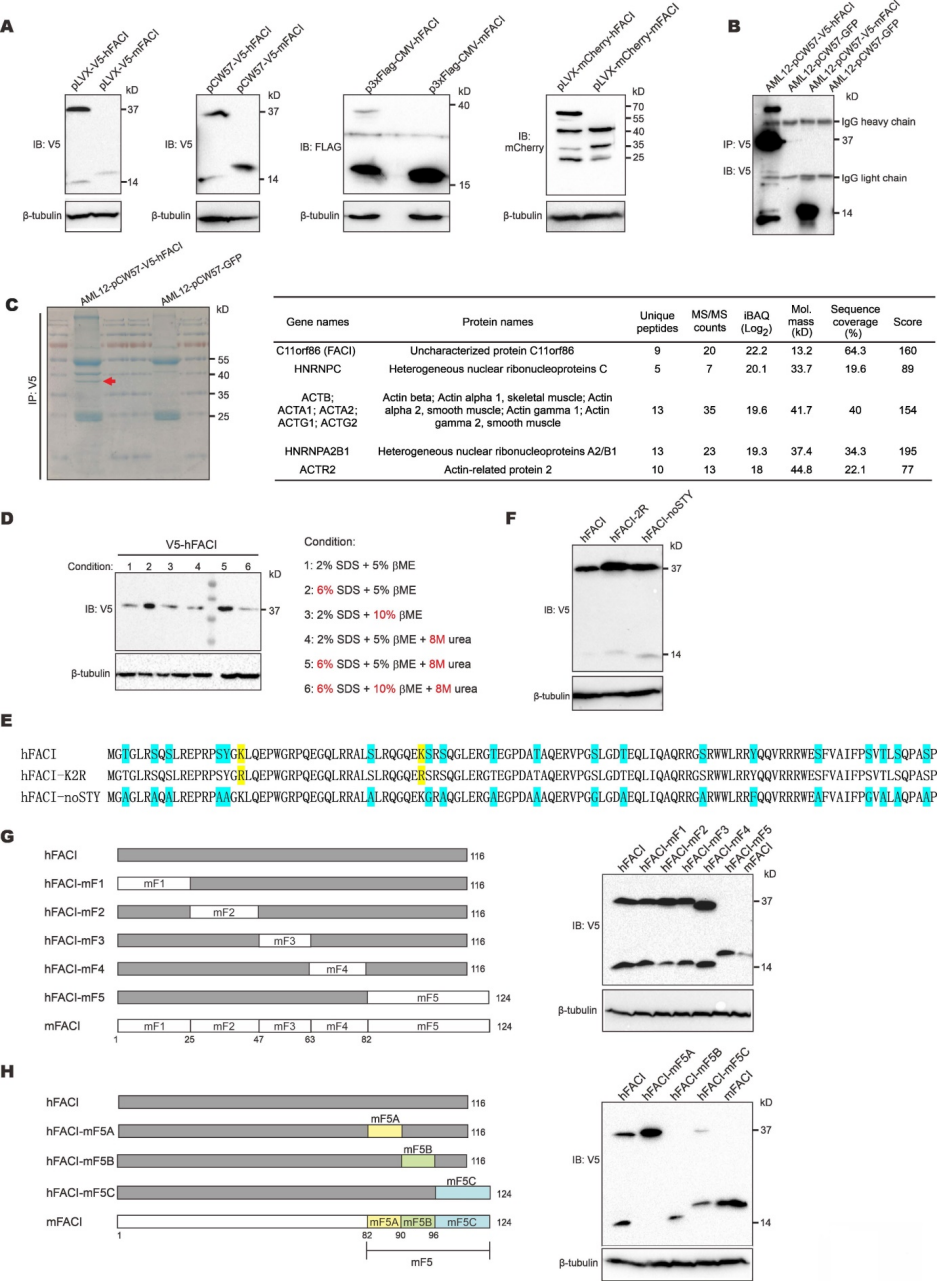
** Appearance of an extra 37-kD protein band in SDS-PAGE analysis of lysates of cells transfected with an expression vector for human FACI.** (**A**) Immunoblotting. HEK293T cells were transfected with the indicated hFACI or mFACI expression plasmids. After 48 hours, total proteins were extracted, separated by SDS-PAGE and probed with the indicated antibodies. (**B**) Co-immunoprecipitation. AML12 cells stably expressing V5-hFACI and V5-mFACI were immunoprecipitated with anti-V5. The immunoprecipitates were lysed and analyzed by immunoblotting. Anti-V5 was used as the probe. Stable cell lines were generated with pCW57-V5-hFACI and pCW57-V5-mFACI. The AML12 cell line stably expressing GFP was used as a negative control. (**C**) Immunoprecipitates derived from AML12-pCW57-V5-hFACI and AML12-pCW57-GFP stable cell lines obtained in (A) were separated by SDS-PAGE and visualized by Coomassie blue staining. The 37-kD band indicated by a red arrowhead was collected for mass spectrometric analysis (left). Proteins identified by mass spectrometry were listed according to protein intensity (i.e., iBAQ value) (right). (**D**) Immunoblotting. HEK293T cells were transfected with pLVX-V5-hFACI plasmid for 2 days. Cell were lysed and proteins were denatured and reduced with various conditions as indicated. Protein samples were then separated by SDS-PAGE and probed with anti-V5. (**E**) Two hFACI mutants, hFACI-K2R and hFACI-noSTY, were generated and cloned into pLVX-Puro vector with N-terminal V5 tag. Protein sequences of hFACI-K2R and hFACI-noSTY mutants were illustrated. (**F**) Immunoblot results of hFACI (WT), hFACI-K2R and hFACI-noSTY. (**G**) A list of human-mouse chimeric FACI constructs (left) and their protein expression (right). All chimeric constructs were cloned into the pLVX-Puro vector with N-terminal V5 tag. (**H**) A list of human-mouse chimeric FACI constructs derived from pLVX-V5-hFACI-mF5 (left). Protein expression of these chimeric constructs was detected by immunoblotting (right).

To explore the physiological function of FACI protein, we expressed four distinctly tagged hFACI and mFACI proteins using pLVX-Puro, pCW57-GFP-2A-MCS, p3×Flag-CMV-10 and pLVX-mCherry-C1 vectors. The pLVX-Puro is a lentiviral expression vector with a puromycin resistance marker 19. The pCW57-GFP-2A-MCS is an all-in-one doxycycline inducible lentiviral vector with a puromycin resistance marker 20. The p3×Flag-CMV-10 is a protein expression vector with a neomycin resistance marker 21. The pLVX-mCherry-C1 is a lentiviral vector for expression of mCherry-tagged fusion protein 19. The predicted sizes of hFACI and mFACI are 14 kD and 15 kD, respectively. In addition to the expected hFACI band of 14 kD, an extra 37-kD band appeared when hFACI was expressed from pLVX-V5-hFACI, pCW57-V5-hFACI, and p3×Flag-CMV-hFACI, but the band was not seen when mFACI was expressed from the same vectors (Figure 1A). Likewise, in addition to the expected mCherry-hFACI species of 40 kD, an extra 60-kD band was also observed when pLVX-mCherry-hFACI but not pLVX-mCherry-mFACI was the expression vector. Consistent with results from transient transfection, the extra 37-kD band was also evident when hFACI was stably expressed from cell lines carrying pLVX-V5-hFACI and pCW57-V5-hFACI (Supplementary Figure S1A).

To reveal the identity of the 37-kD band, we immunoprecipitated V5-hFACI protein from AML12-V5-hFACI stable cell line (Figure 1B) and collected the 37-kD band for mass spectrometric analysis (Figure 1C, left). Proteins identified by mass spectrometry were listed next to the gel image in Figure 1C. Among the identified proteins, hFACI had the highest protein intensity as judged by the intensity- based absolute quantitation (iBAQ) value, which indicated that the 37-kD band was derived from hFACI protein per se.

Protein electrophoretic mobility shifts are usually caused by noncovalent or covalent homo/hetero-protein interactions and PTMs 14, 15. To disrupt all potential noncovalent bonds between proteins, we lysed protein samples with harsh denaturing lysis buffers containing urea and 3× SDS. We also lysed protein samples with 2× β-mercaptoethanol (βME) to ensure complete reduction of disulfide bonds. Under all conditions for sample lysis listed next to the immunoblot, the 37-kD band remained unchanged (Figure 1D). In addition, no bona fide FACI-interacting partner proteins were identified in further analysis of the mass spectrometric results (Figure 1C). Therefore, the 37-kD band was unlikely due to protein interaction.

Ubiquitination, sumoylation and glycosylation can also alter protein size to great extent, leading to substantial gel shifts in SDS-PAGE. Classic ubiquitination and sumoylation occur on lysine (K) residues. N-linked glycosylation occurs on asparagine (N), while O-linked glycosylation occurs on serine (S), threonine (T) and tyrosine (Y) 14. To explore whether these PTMs might account for the 37-kD band of hFACI, we mutated all target residues of ubiquitination, sumoylation and O-linked glycosylation on hFACI. N-linked glycosylation was not considered since N residues are absent in hFACI. Two hFACI mutants were generated (Figure 1E). The hFACI-K2R was designed to preclude ubiquitination and sumoylation on K residues. The hFACI-noSTY, of which S, T and Y residues had been eliminated, was used to block O-linked glycosylation. It is noteworthy that phosphorylation was also prevented in this mutant. Immunoblotting results indicated both K2R and noSTY mutants had strong 37-kD bands (Figure 1F), suggesting that the 37-kD band was unlikely caused by classical PTMs.

To narrow down the protein region in hFACI that gives rise to the extra 37-kD band, we designed a series of chimeric FACI constructs on pLVX-Puro backbone. Human and mouse FACI were divided into 5 fragments (F1 to F5) and different sections were joined together as shown in Figure 1G and Supplementary Figure S1B. Immunoblot results indicated disappearance of the 37-kD band upon expression of hFACI-mF5 with human fragments 1 to 4 plus mouse fragment 5 (Figure 1G), while the 37-kD band remained prominent when hFACI-mF1-4 carrying mouse fragments 1 to 4 plus human fragment 5 was expressed (Supplementary Figure S1B). Thus, hFACI fragment 5 might account for the 37-kD band shift. We further designed another series of chimeric constructs derived from hFACI-mF5 (Figure 1H) and hFACI-mF1-4 (Supplementary Figure S1C). The 37-kD bands disappeared when the expression of hFACI-mF5B, hFACI-mF1-5B and hFACI-m1-116 was enforced (Figure 1H and Supplementary Figure S1C). That is to say, a critical site present in hF5B but absent in mF5B is required for the formation of the extra 37-kD protein band.

### The unusual 37-kD band derived from recombinant hFACI results from aberrant splicing

**Figure 2:**
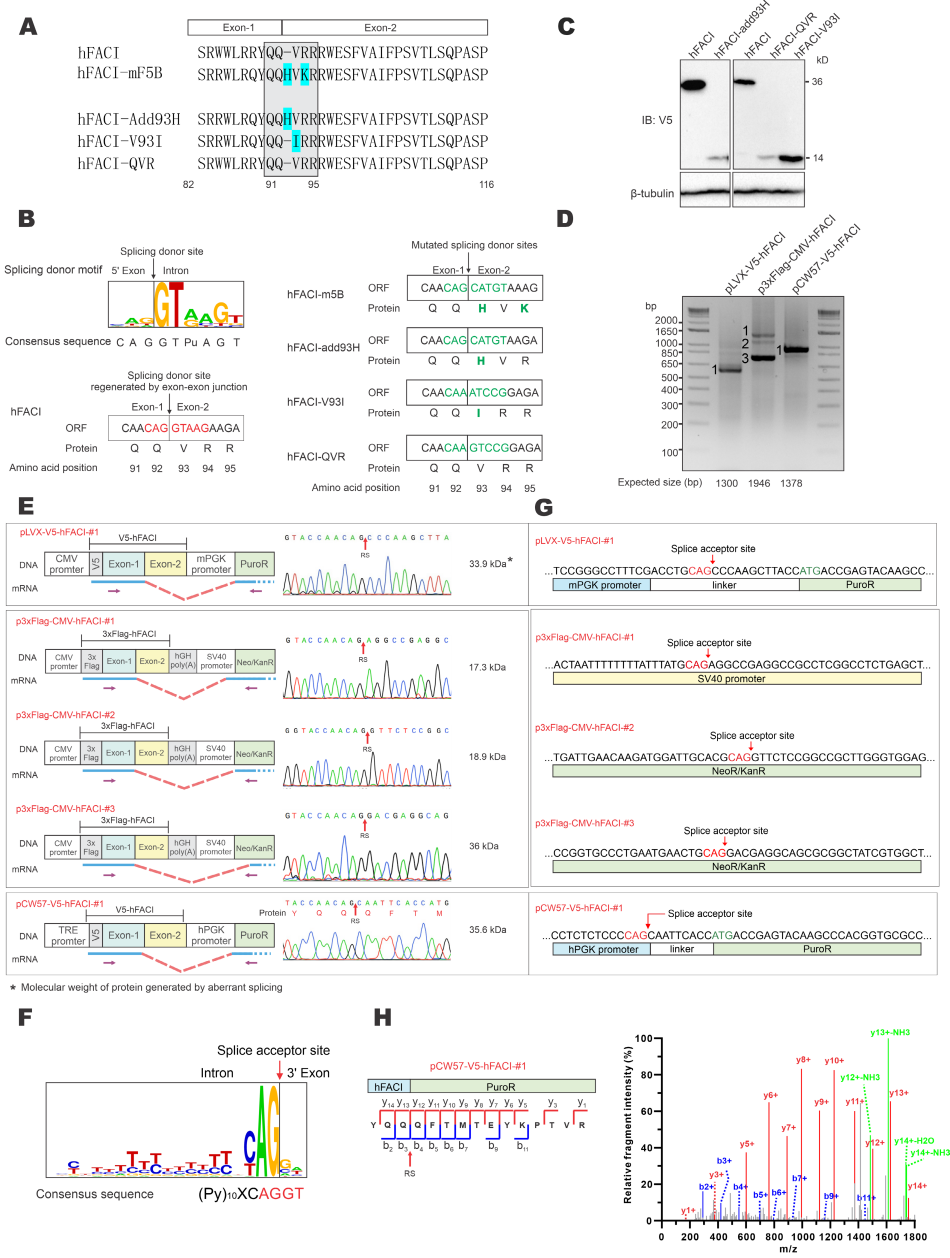
**The extra 37-kD protein band of hFACI was generated by aberrant splicing.** (**A**) Protein sequence of hFACI, hFACI-mF5B and other three mutants. The EJ of hFACI ORF happens to locate at the sequence encoding “QQ|VRR” (boxed). hFACI-QVR is a version of hFACI in which anonymous mutations had been introduced to the codons of “QVR”. The amino acid position was based on hFACI. All mutants were constructed into pLVX-Puro with a N-terminal V5 tag. (**B**) The EJ sequence of hFACI “CAG|GTAAG” (in red) exactly matches the consensus sequence of human splice site (left). The “splice donor-like” EJ sequence (in green) was disrupted in hFACI-m5B, and other three mutants (right). Human splice site consensus sequence has been reported previously 4, 27. (**C**) Immunoblot results revealed disappearance of the extra 37-kD band in HEK293T cells expressing hFACI-add93H, hFACI-QVR or hFACI-V93I. (**D**) RT-PCR results. HEK293T cells were transfected with pLVX-V5-hFACI, p3×Flag-CMV-hFACI or pCW57-V5-hFACI for 36 hours. Total RNA was extracted and the human *FACI* transcripts were amplified by RT-PCR. Selected PCR bands were further verified by DNA sequencing. Expected sizes of the PCR fragments derived from unspliced transcripts were also listed below the gel image. Fragments of smaller sizes were highlighted by numbers 1 and 2. (**E**) Schematic diagrams depicting aberrant splicing events on pLVX-V5-hFACI, p3×Flag-CMV-hFACI, and pCW57-V5-hFACI. PCR bands derived from aberrant splicing of human *FACI* in (D) were sequenced. The positions of sequencing primers were indicated with purple arrows in the diagrams. The predicted ORF sizes from the spliced transcripts were also listed. The corresponding sequencing chromatograms were also shown. The molecular weight of the protein product generated by aberrant splicing was highlighted with an asterisk (*). RS: the site of splicing junction. CMV: cytomegalovirus. hPGK: human phosphoglycerate kinase. PuroR: puromycin resistance. hGH: human growth hormone. Neo/KanR: neomycin-kanamycin resistance. TRE: tetracycline response element. mPGK: mouse phosphoglycerate kinase. (**F**) The sequence logo of human splice acceptors. Consensus sequence of human splice sites has been described 4, 27. (**G**) The splice acceptor sites identified on pLVX-V5-hFACI, pCW57-V5-hFACI and p3×FLAG-CMV-10-hFACI by RT-PCR and sequencing. (**H**) One MS/MS spectrum of the junction peptide of the hFACI-PuroR fusion protein was provided. Method: collision-induced dissociation time-of-flight MS.

Between hF5B and mF5B, just one small region was different (Figure 2A, upper). The protein sequence of hFACI 92-94 is “QVR”, while the compartment in mFACI is “QHVK”. Interestingly, the coding sequence for “QVR” happens to lie within the EJ or splice junction of human *FACI* ORF. For simplicity and ease of direct comparison, hereafter we will use the DNA sequence cognate to mRNA to describe EJ and splice sites. We found the EJ sequence “CAG|GTAAG” in the human *FACI* gene exactly matches the consensus sequence of splice donor, but this sequence was disrupted in hFACI-m5B (Figure 2A-B).

To verify whether this splice donor site of hFACI might contribute to an unexpected mRNA splicing event, giving rise to an aberrant 37-kD protein, three mutants were constructed: hFACI-add93H, hFACI-V93I and hFACI-QVR (Figure 2A, bottom). In hFACI-add93H, a histidine residue was inserted between Q92 and V93. In hFACI-V93I, the valine residue at position 93 was mutated to isoleucine. And in hFACI-QVR, the QVR residues were silently mutated to eliminate the potential splicing donor sequence. The splice donor sites within the EJs were disrupted in all three mutants. Detailed DNA and amino acid sequences of the three mutants could be found in Figure 2B. Immunoblot results demonstrated the loss of the 37-kD band when hFACI-add93H, hFACI-QVR or hFACI-V93I was expressed in HEK293T cells (Figure 2C), lending support to the notion that the 37-kD band results from unexpected splicing.

Next, we further confirmed the occurrence of the aberrant splicing event when hFACI was expressed from pLVX-V5-hFACI, pCW57-V5-hFACI and p3×Flag-CMV-hFACI. RT-PCR was performed to investigate the formation of fusion transcripts in which *FACI* sequence was aberrantly spliced to downstream sequence on the plasmid. The sizes of the one or two amplified fragments derived from RNA transcripts expressed from pLVX-hFACI, pCW57-hFACI and p3×Flag-CMV-hFACI were all smaller than expected (Figure 2D). Thus, aberrant splicing was detected when hFACI was expressed from all three constructs.

The RT-PCR products were further sequenced to determine the splice donor and acceptor sites. The sequencing results of splice junctions together with schematic diagrams depicting the splicing events were presented in Figure 2E. The splice donor sites were all on the expected EJs in the three hFACI constructs (Figure 2E). The splice acceptors were contributed by distinct plasmid sequences (Figure 2E-F). For pLVX-V5-hFACI and pCW57-V5-hFACI, the acceptor site lies on the linker sequence between phosphoglycerate kinase (PGK) promoter and puromycin resistance (PuroR) gene. It is noteworthy that the linker sequences are different for pLVX- and pCW57-backbone. The PGK promoters of pLVX- and pCW57-backbone are also from different species. For p3×Flag-CMV-hFACI, two aberrantly spliced transcripts were identified. Their splicing acceptors were located on the SV40 promoter and neomycin/kanamycin resistance (Neo/KanR) gene, respectively (Figure 2G).

We also re-analyzed the raw data of mass spectrometric experiment performed in Figure 1C and found the tandem mass spectrometry (MS/MS) spectrum of the junction peptide (“YQQ|QFTMTEYK…”) of hFACI-PuroR (Figure 2H). This MS/MS spectrum had extremely high intensity as well as a high-confidence score, which further verified that the 37-kD band represents a fusion protein of 15-kD V5-hFACI and 22-kD PuroR.

### Whole genome search of genes susceptible to vector-dependent aberrant splicing

Above we demonstrated aberrant splicing ascribed to a cryptic splice donor site on the EJ of human *FACI* after its insertion into an expression vector. This should not be a single case. Other genes carrying EJs that match the splice donor sequence are also at high risk of undergoing vector-dependent aberrant splicing during overexpression. We performed whole genome analysis on the DNA sequence cognate to mRNA to search for these genes among all protein-coding genes. First, genes with an EJ sequence of “N|GT'' were selected, since around 99% of splice donor sites have “N|GT''. Human and mouse genes susceptible to vector-dependent aberrant splicing based on this criterion were listed in Supplementary Tables S2 and S3, respectively. Second, a more stringent criterion was used to select genes that carry an EJ sequence of “AN|GTpuAG”, because “AN|GTpuAG” is the most common and conserved splice site. Human and mouse genes susceptible to vector-dependent aberrant splicing based on this stringent criterion were summarized in Supplementary Tables S4 and S5, respectively. From 20,394 human protein-coding genes, 11,926 and 623 genes that satisfy the above two criteria were identified. The equivalent three numbers in mice were 17,056, 9,881 and 506.

### Aberrant splicing frequently occurs during foreign gene expression

**Figure 3:**
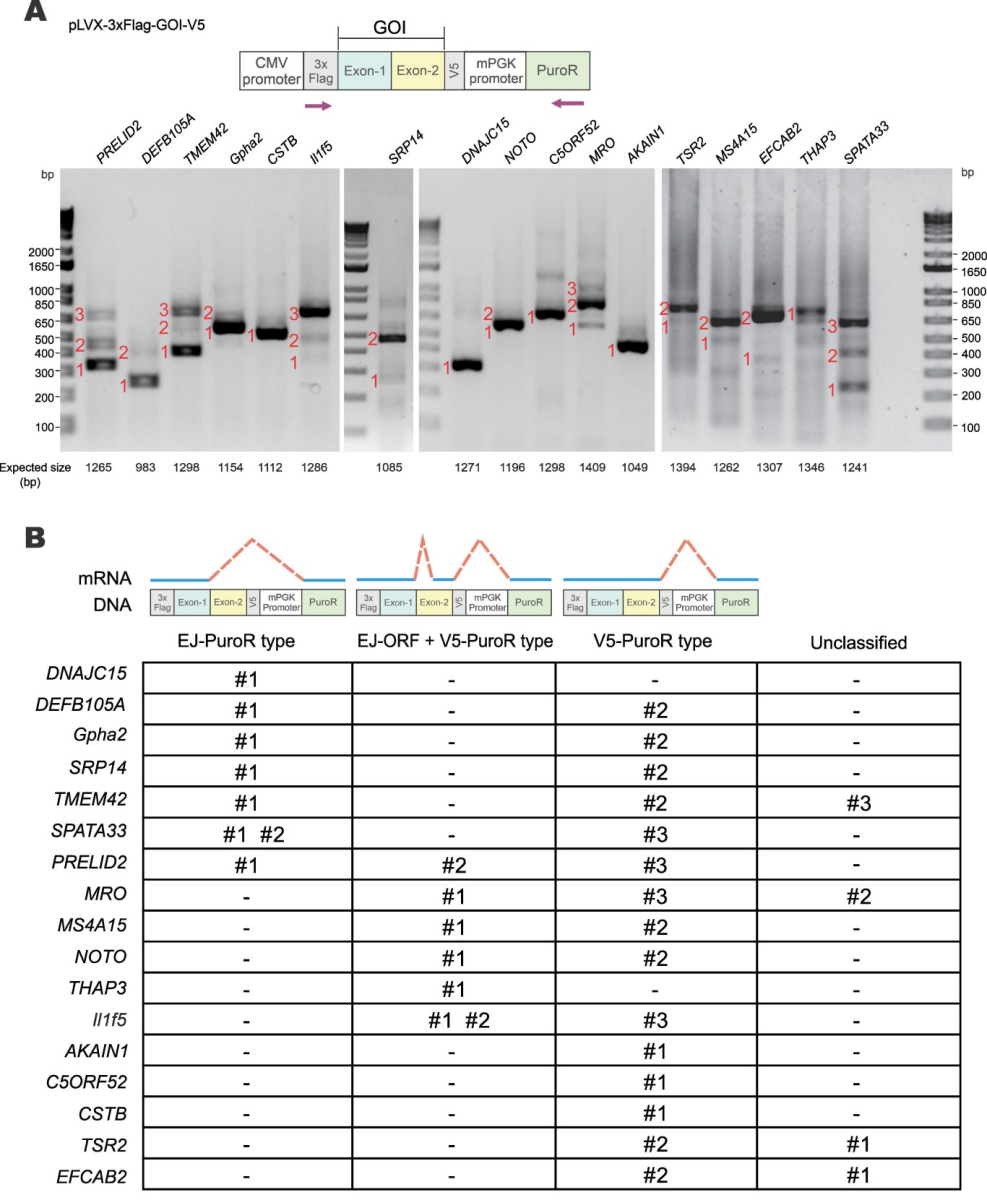
**Verification of vector-dependent aberrant splicing in all 17 selected genes.** (**A**) A total of 17 human and mouse genes predicted to be susceptible to vector-dependent aberrant splicing were selected and cloned into pLVX-Puro vector with an N-terminal 3×Flag tag and a C-terminal V5 tag. The plasmid was transfected into HEK293T cells. After 36 hours, total RNA was extracted, and gene transcripts were amplified by RT-PCR. The positions of sequencing primers used were highlighted in the schematic diagram of the construct. Expected molecular sizes of PCR fragments derived from unspliced transcripts were also provided under the gel panels. Fragments of smaller sizes were highlighted by numbers 1, 2 and 3 in red. RT-PCR reactions performed in the absence of reverse transcriptase yielded no band. CMV: cytomegalovirus. mPGK: mouse phosphoglycerate kinase. PuroR: puromycin resistance. (**B**) Selected PCR bands derived from aberrant splicing of the 17 genes in (**A**) were sequenced. Based on the sequencing results, aberrant splicing events seen in the 17 genes were classified into 4 types: EJ-PuroR, EJ-ORF + V5-PuroR, V5-PuroR and unclassified.

To verify how common vector-dependent aberrant splicing would be, 17 human and mouse genes identified by stringent criterion were randomly selected for experimental validation (Supplementary Table S6). The selected GOIs were cloned into pLVX-Puro vector with both N-terminal 3×Flag and C-terminal V5 tags (Figure 3A). HEK293T cells were transfected with pLVX-3×Flag-GOI-V5 constructs and transcripts of GOIs were examined by RT-PCR. Sizes of all PCR products of the GOI transcripts were smaller than expected, indicating the occurrence of aberrant splicing (Figure 3A). PCR products numbered in Figure 3A were further cloned into pGEM-T vector and sequenced to determine the splice donor and acceptor sites. Sequencing chromatograms of splice donor sites of 17 GOIs were presented in Supplementary Figure S2 and S3.

Three major types of aberrant splicing were identified and summarized in Figure 3B. They are EJ-PuroR, EJ-ORF + V5-PuroR, and V5-PuroR types. For the EJ-PuroR type, splice donor sites are located at the EJs, while acceptor sites are on the PuroR selectable marker or the linker sequence between the mouse PGK (mPGK) promoter and PuroR. EJ-PuroR-type splicing was found in *DNAJC15*, *DEFB105A*, *Gpha2*, *SRP14*, *TMEM42*, *SPATA33* and *PRELID2* genes. EJ-ORF + V5-PuroR type refers to two aberrant splicing events that occurred on one pre-mRNA. For the first splicing event, the donor site is located at the EJ, while the acceptor site is a cryptic one within the ORF region of a GOI. The second splicing event involves a donor site located surprisingly at the V5 tag sequence and an acceptor site found within the linker sequence between the mPGK promoter and PuroR. EJ-ORF + V5-PuroR type aberrant splicing was found in *PRELID2*, *MRO*, *MS4A15*, *NOTO*, *THAP3* and *Il1f5* genes. For the V5-PuroR type, the splicing donor site is within the V5 tag sequence, while the acceptor site is on the aforementioned linker sequence. Aberrant splicing of the V5-PuroR type was identified in 16 GOIs except in *THAP3* genes. Although it was not identified in *DNAJC15* gene by RT-PCR, the V5-PuroR type of aberrant splicing did occur in *DNAJC15* according to the immunoblot results to be discussed later (Figure 5B).

Besides the three major types, other aberrant splicing events were also found in *MRO*, *TMEM42*, *TSR2* and *EFCAB2* genes and they were grouped into the unclassified type (Figure 3B). For *TSR2*-#1 and *EFCAB2*-#1, the splice donor sites are the cryptic splice donor sites on their ORFs. The acceptor sites are located at or near PuroR. *TMEM42*-#3 and *MRO*-#2 transcripts are aberrantly spliced twice. For *TMEM42*-#3, the donor and acceptor sites of the first splicing event are located at the V5 tag and mPGK promoter, respectively. The splice sites of the second splicing event are within mPGK promoter and PuroR. For *MRO*-#2, the splice sites of the first splicing event are respectively located at V5 tag and PuroR. Both donor and acceptor sites of the second splicing event are within PuroR (Supplementary Figure S2, Supplementary Figure S3).

**Figure 4:**
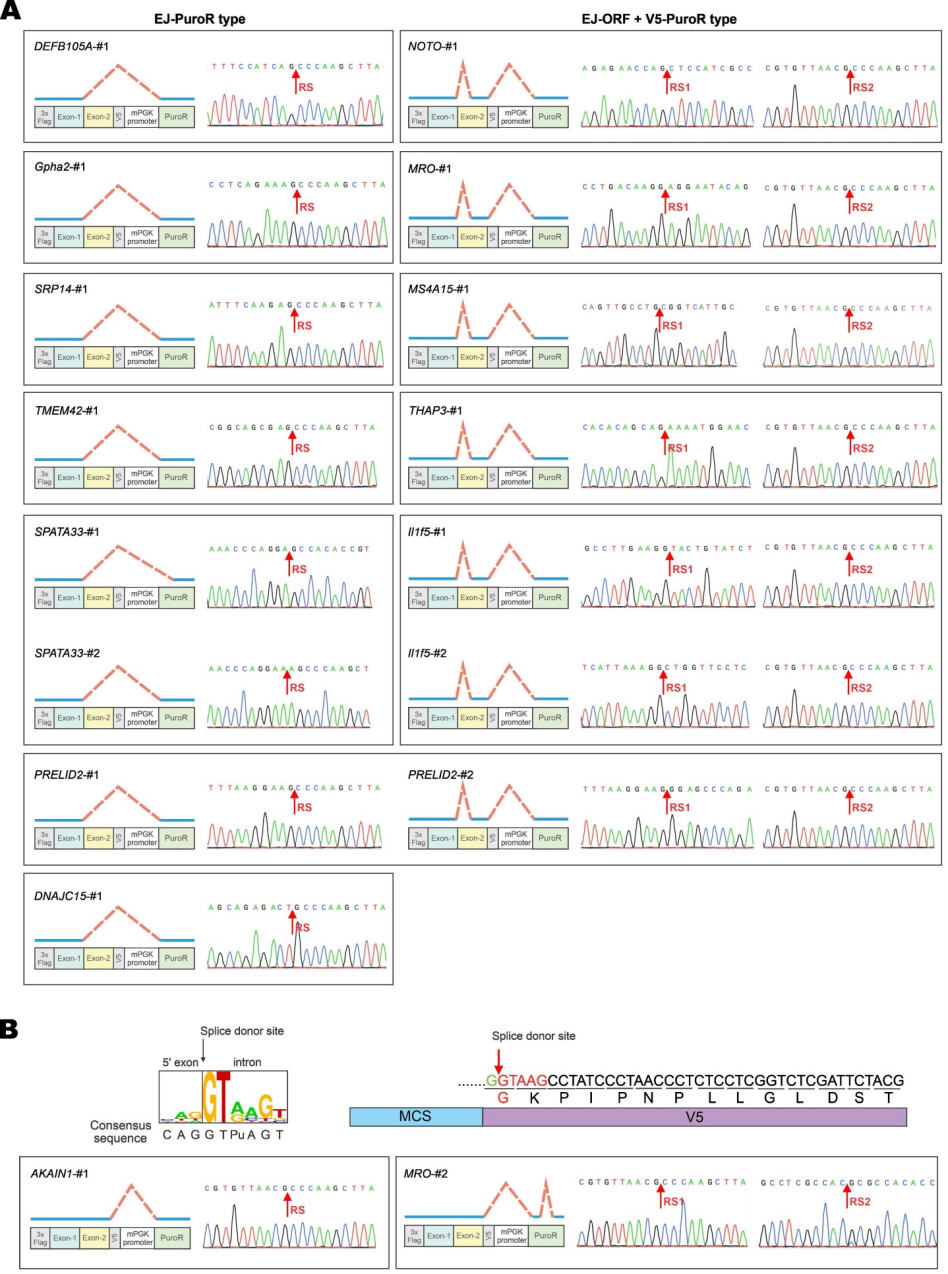
**Further verification of vector-dependent aberrant splicing in selected genes.** (**A**) Schematic diagrams and sequencing chromatograms of EJ-PuroR-type and EJ-ORF + V5-PuroR-type aberrant splicing. In these two types of aberrant splicing, the EJs serve as the splice donors. (**B**) Schematic diagrams of the consensus sequence of human splice donors 4, 27, and the splice donor sequence within V5 tag (top). V5-derived aberrant splicing was illustrated with the examples of AKAIN1-#1 and MRO-#2 (bottom). RS: the site of splicing junction.

Among the 17 GOIs, 13 underwent aberrant splicing of the EJ-PuroR or EJ-ORF + V5-PuroR type. Both types recognize EJ sequences of GOIs as splice donors, indicating frequent occurrence of aberrant splicing in the GOIs. The schematic diagrams and sequencing chromatograms of EJ-PuroR and EJ-ORF + V5-PuroR types of aberrant splicing were presented in Figure 4A.

During the analysis of the splice donor sites, we found that the “G|GTAAG” sequence in V5 tag serves as a splice donor site (Figure 3B, Figure 4B). The “G|GTAAG”-containing sequence encoding V5 tag is widely used in commercial vectors. Representative schematic diagrams and sequencing chromatograms of V5 tag-mediated aberrant splicing were presented in Figure 4B. Aberrant splicing on V5 tag occurred in all 17 cases. This indicated that the “G|GTAAG” of V5 tag sequence is a robust splice donor site, raising concern for the use of V5 tag.

In addition, at least four splice acceptor sites were present in pLVX-Puro vector (Supplementary Figure S4). The linker sequence between the mPGK promoter and PuroR was the most robust one and mediated most aberrant splicing events seen upon expression of the GOIs.

### Vector-mediated aberrant splicing results in fusion protein expression

To verify fusion protein expression resulting from vector-dependent aberrant splicing, we selected 10 genes in Supplementary Table S6 for further analysis. For each GOI, three types of mutants were generated: R-EJ, R-V5 and R-EJ-V5. The donor sequences on EJ and V5 tag were synonymously mutated in the R-EJ and R-V5 mutants, respectively. Both donor sequences were synonymously inactivated in the R-EJ-V5 mutant.

**Figure 5:**
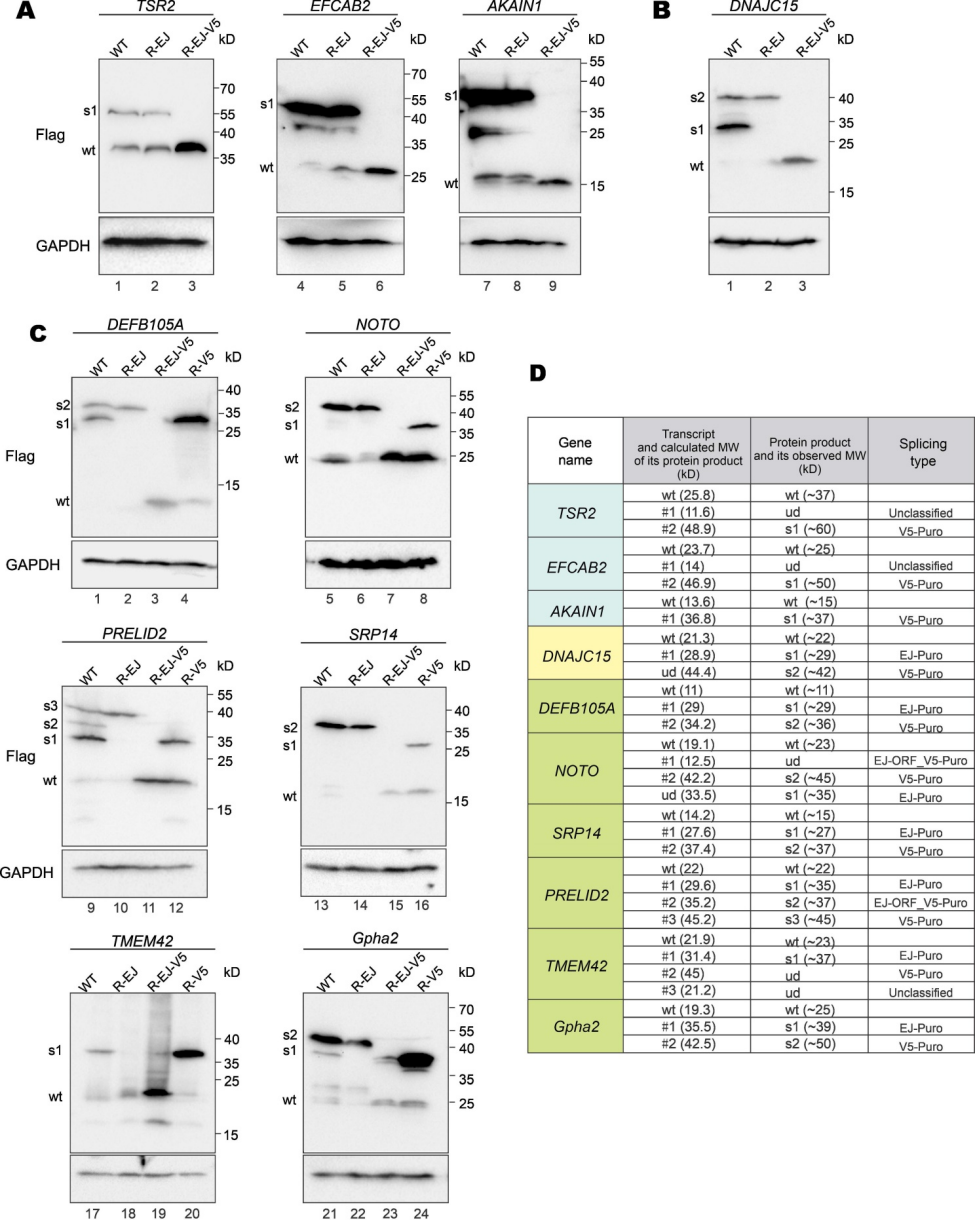
**Protein expression from aberrantly spliced transcripts.** (**A**) Immunoblot and mutational analysis of *TSR2*, *EFCAB2* and *AKAIN1* genes of the V5-PuroR-type. In the R-EJ-mutant, the splice donor sequence on the EJ was synonymously mutated. In the R-EJ-V5-mutant, both the donor sequence on the EJ and V5 tag was synonymously removed. (**B**) Immunoblot and mutational analysis of *DNAJC15* of the EJ-PuroR-type. However, the V5-PuroR-type aberrant splicing was also present as indicated by immunoblotting. (**C**) Immunoblot and mutational analysis of additional six genes (*Gpha2*, *NOTO*, *PRELID2*, *SRP14*, *TMEM42* and *SPATA33*). Both the EJs and V5 tags of all 6 genes serve as splice donor sites according to RT-PCR. The splice donor sequence within V5 tag was synonymously mutated in the R-V5-mutant. (**D**) Correlation of aberrantly spliced transcripts (Figure 3B) and their protein products (Figure 5A to 5C). Theoretical and actual molecular weight (MW) of protein products of aberrantly spliced transcripts were compared and summarized. wt: wild type. s1-3: splice isoforms 1-3. ud: undetermined.

The RT-PCR and sequencing results indicated that aberrant splicing occurs at the V5 tag but not the EJ (Figure 3B) in the cases of *AKAIN1*, *TSR2* and *EFCAB2* genes. Therefore, protein expression of wild-type (WT), R-EJ mutant and EJ-V5 mutant were analyzed. The R-EJ mutant and WT of each protein showed the same immunoblot pattern (Figure 5A), indicating the lack of aberrant splicing involving the EJ. However, in the R-EJ-V5 mutant of each protein, the upper band disappeared but the band of the expected molecular mass retained. These results were consistent with the occurrence of aberrant splicing within the V5 tag sequence. Although the cryptic splice donor sites in *TSR2* and *EFCAB2* genes also fueled the aberrant splicing (Figure 3B), no visible protein bands were seen in the immunoblots. This was not surprising since the fusion transcripts arisen from such aberrant splicing (i.e., TSR2-#1 and EFCAB2-#1) were barely detectable as shown in Figure 3A.

In the earlier RT-PCR result of *DNAJC15*, only EJ-PuroR type of aberrant splicing was observed (Figure 3B). According to the immunoblot result (Figure 5B), recoding the EJ using synonymous mutations in the R-EJ mutant only led to the elimination of the 30-kD protein band. However, in the R-EJ-V5 mutant, both the 30-kD and the 40-kD bands disappeared and the 19-kD band of the expected molecular mass retained. This result suggested that *DNAJC15* undergoes both EJ-derived and V5-derived aberrant splicing, although the cDNA band of the V5-derived aberrant splicing was too weak to be detected by RT-PCR.

*Gpha2*, *NOTO*, *PRELID2*, *SRP14*, *TMEM42* and *DEFB105A* genes were determined to undergo both EJ-derived and V5-derived aberrant splicing (Figure 3B). Compared with the R-EJ-V5 mutants of 6 GOIs, all their R-V5 mutants exhibited several extra protein bands (Figure 5C, lanes 3, 4, 7, 8, 11, 12, 15, 16, 19, 20, 23 and 24). The results indicated the occurrence of EJ-derived aberrant splicing in all 6 GOIs. Likewise, the R-EJ mutants of *Gpha2*, *NOTO*, *PRELID2*, *SRP14* and *DEFB105A* genes also demonstrated significantly different protein bands with their R-EJ-V5 counterparts in the immunoblot assays (Figure 5C, lanes 2, 3, 6, 7, 10, 11, 14, 15, 22 and 23). The results indicated occurrence of V5-derived aberrant splicing in those GOIs. Compared with the R-EJ-V5 mutant of *TMEM42*, extra protein bands could not be observed in the R-EJ mutant (Figure 5C, lanes 18 and 19). One possible reason is that the transcript generated by V5-derived aberrant splicing (i.e., TMEM42-#2) was too weak (Figure 3A), leading to no detection of translated protein by immunoblotting.

We further analyzed whether observed protein bands in immunoblots (Figure 5A to 5C) were consistent with aberrantly spliced transcripts in RT-PCR (Figure 3A and 3B). As indicated in Figure 5D, for most transcripts, the molecular weight (MW) of their actual protein products was compatible with the MW of their theoretical protein products. Five transcripts (TSR2-#1, EFCAB2-#1, NOTO-#1, TMEM42-#2 and TMEM42-#3) didn't generate visible protein bands. TSR2-#1, EFCAB2-#1 and TMEM42-#2 transcripts were in low abundance, and their protein products were hardly detectable by immunoblotting. NOTO-#1 and TMEM42-#3 contain premature translation termination codons and were probably cleared by nonsense-mediated mRNA decay (NMD) before translation. Additionally, two protein bands (DNAJC15-s1 and NOTO-s1) were observed but the cognate aberrantly spliced transcripts were not identified by RT-PCR. NOTO-s1 was derived from EJ-Puro aberrant splicing. In NOTO WT, the EJ-Puro aberrant splicing is inefficient. Both transcript and protein of NOTO-s1 were weak and hardly visible. However, in NOTO R-V5 mutant, removal of the V5-splicing donor site greatly increased the efficiency of EJ-Puro aberrant splicing, enabling strong expression of NOTO-s1. DNAJC15-s1 is derived from V5-Puro aberrant splicing. We failed to acquire the transcript for DNAJC15-s1 because of its low abundance.

**Figure 6:**
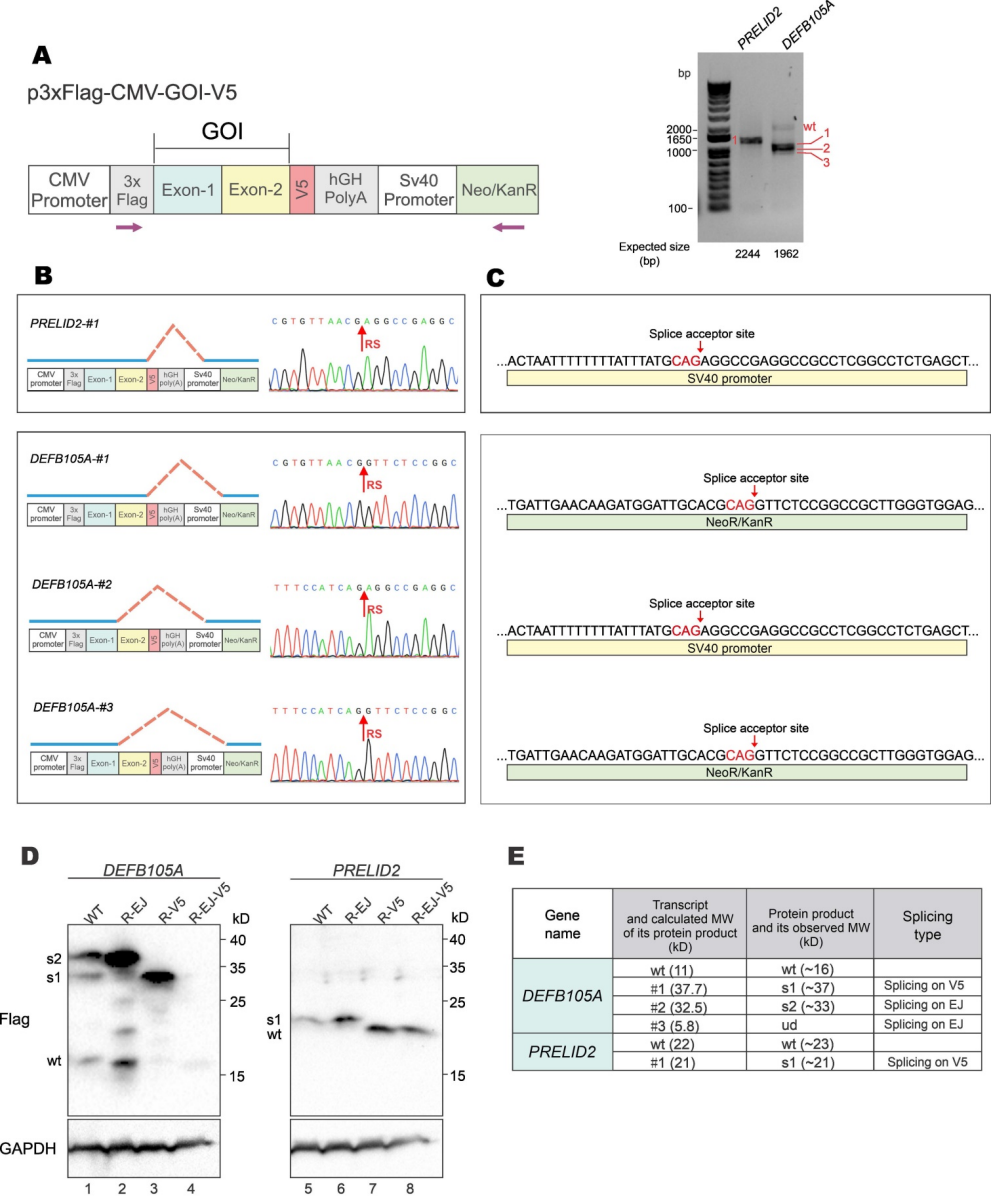
** Aberrant splicing and protein expression from p3×FLAG-CMV-10 vector.** (**A**) DEFB105A and PRELID2 ORFs were cloned into p3×FLAG-CMV-10 vector with a C-terminal V5 tag. The plasmid was transfected into HEK293T cells. After 36 hours, total RNA was extracted, and gene transcripts were amplified by RT-PCR. The positions of sequencing primers used were indicated in the schematic diagram of the construct (left). Expected sizes of PCR fragments derived from unspliced transcripts were provided under the gel panels. Fragments of smaller sizes were highlighted by numbers 1, 2 and 3 in red (right). CMV: cytomegalovirus. hGH: human growth hormone. Neo/KanR: neomycin-kanamycin resistance. (**B**) Schematic diagrams depicting aberrant splicing events on p3×Flag-CMV-DEFB105A and p3×Flag-CMV-PRELID2. PCR bands derived from aberrant splicing in (A) were sequenced. The corresponding sequencing chromatograms were also shown. RS: the site of splicing junction. (**C**) The splice acceptor sites identified by RT-PCR and sequencing on p3×Flag-CMV-DEFB105A and p3×Flag-CMV-PRELID2. (**D**) Immunoblot and mutational analysis of DEFB105A and PRELID2 proteins expressed from the p3×FLAG-CMV-10 vector. (**E**) The correlation of aberrantly spliced transcripts (Figure 6B) and their potential protein products (Figure 6D) were examined and analyzed. Theoretical and actual molecular weight (MW) of protein products of aberrantly spliced transcripts were compared and summarized. wt: wild type. s1-3: splice isoforms 1-3. ud: undetermined.

To verify the formation and expression of aberrant fusion proteins when GOIs were expressed from a different vector backbone, GOI expression constructs were made with the p3×FLAG-CMV-10 vector (Figure 6A, left). The p3×FLAG-CMV-10 plasmid contains a poly(A) signal from human growth hormone (hGH), which should avoid transcriptional readthrough into vector sequence. However, RT-PCR results showed that both *DEFB105A* and *PRELID2* had spliced forms of transcripts (Figure 6A, right). Sequencing results further revealed the donor sites of aberrantly spliced *DEFB105A* and *PRELID2* were on the EJ and V5-tag, and the acceptor sites were on SV40 promoter and Neo/KanR gene (Figure 6B and 6C). DEFB105A and PRELID2 proteins expressed from p3×FLAG-CMV-10 were examined by immunoblotting (Figure 6D and Supplementary Figure S5). While *DEFB105A* exhibited both EJ- and V5-derived aberrant splicing, only V5-derived splicing was observed for *PRELID2*. In addition, the protein bands of DEFB105A and PRELID2 were exactly as predicted from their aberrantly spliced transcripts shown in the RT-PCR results (Figure 6E). These results indicated the occurrence of aberrant splicing even though a poly(A) signal is present in the vector.

All sequence information of proteins generated by aberrant splicing was listed in Supplementary Table S7.

### Vector-dependent aberrant splicing frequently occurs in many commercial vectors

**Figure 7:**
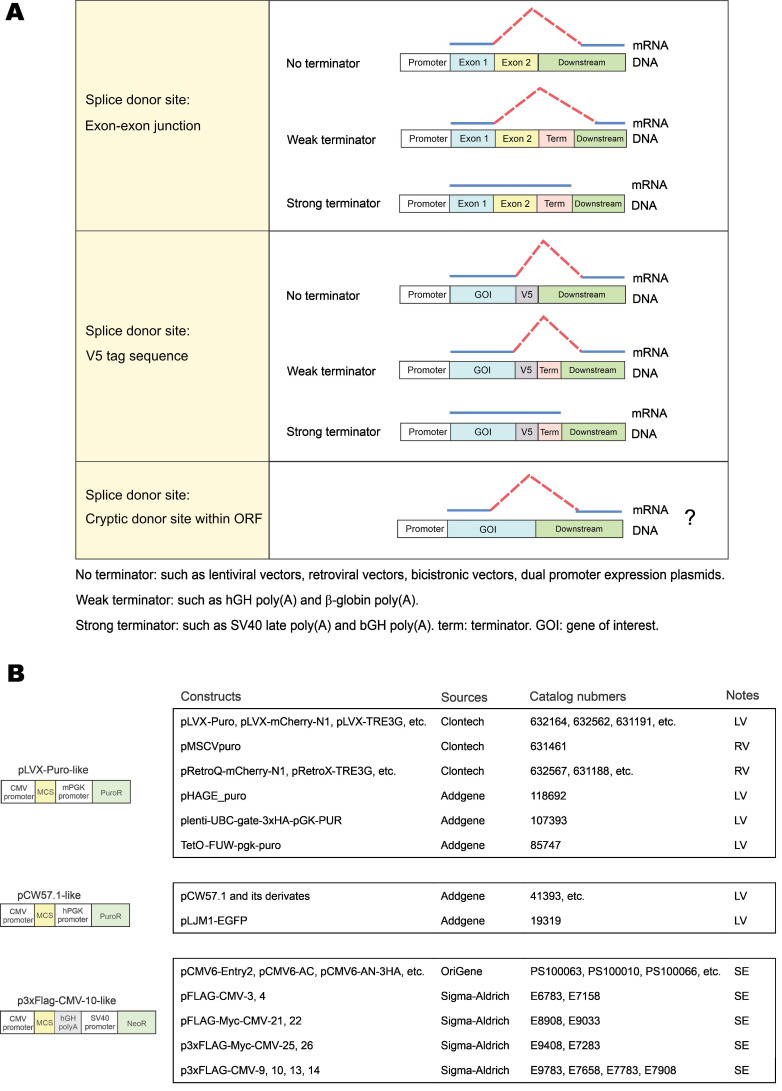
** Three types of vector-dependent aberrant splicing.** (**A**) Summary of three types of vector-dependent aberrant splicing which potentially occurs during the process of foreign gene expression. (**B**) Lists of expression vectors prone to aberrant as identified by sequence similarity analysis. LV: lentiviral vector; RV: retroviral vector; SE: stable expression vector.

In this study, we identified three types of vector-dependent aberrant splicing events that occur when foreign GOIs are inserted into expression vectors (Figure 7A). First, if the EJ sequence of the gene happens to match the splice donor motif, it is likely recognized as a “splice donor site” for aberrant splicing. Second, the V5 tag contains a potential splice donor sequence and, in most cases, could indeed be aberrantly spliced. Third, cryptic splice sites on exons could also be recognized as functional splice donor sites during foreign gene expression (e.g., TSR2-#1 and EFCAB2-#1). Our study also indicated the aberrant splicing on the “splice donor-like” EJ and V5 tag was frequent, while the aberrant splicing on cryptic splice sites within the ORF occurs more rarely. One possible reason is that the essential cis-acting elements adjacent to the EJ efficiently recruit spliceosomes, while the adjacent sequence of the cryptic splice sites normally lacks similar elements to facilitate splicing 22.

The risks of aberrant splicing seem to be not only related with splicing donors, but also with the vectors used for gene expression (Figure 7A). First, if the vector does not provide a transcriptional terminator immediately downstream of the foreign GOI, readthrough transcription proceeds into the vector sequence until a transcriptional terminator is encountered. The vector sequence in the readthrough transcript has a greater chance to provide functional splice acceptor sites in cis to mediate aberrant splicing. Expression systems without immediate downstream transcriptional terminators include lentiviral vectors, retroviral vectors, multicistronic vectors (e.g., IRES- or T2A-containing vectors) and dual promoter expression vectors (e.g., PSF-CMV-PGK-dual promoter expression plasmid from Sigma-Aldrich). In our case, since no terminator was found in lentiviral pLVX-hFACI, PuroR was transcribed to provide several splice acceptors for aberrant cis-splicing in the readthrough transcript (Figure 2G). Second, weak terminators, such as human growth hormone (hGH) and β-globin poly(A) signals, might not be efficiently strong enough to stop transcription of the foreign gene 23. The resulting readthrough transcript has a higher risk of aberrant splicing if splice donors exist on this transcript. In our case of the p3×Flag-CMV-hFACI plasmid, a readthrough hFACI-neomycin fusion transcript was generated and aberrant splicing was mediated by two splice acceptors on Neo/KanR. Third, strong terminators, such as bovine growth hormone (bGH) and SV40 late poly(A) signals, should be sufficient to stop transcription of the foreign GOI. Therefore, theoretically, aberrant splicing involving downstream vector sequence can be avoided.

Apart from pLVX-Puro, pCW57-GFP-2A-MCS and p3×Flag-CMV-10, some other commercial vectors are also prone to vector-dependent aberrant splicing when used for expression of foreign GOI. By sequence similarity analysis, some widely used vectors with high risks of vector-dependent aberrant splicing were categorized in Figure 7B. The pLVX-Puro-like vector contains the same sequence with pLVX-Puro on the mPGK promoter, linker and PuroR. Likewise, the pCW57.1-like vector contains the same sequence with pCW57-GFP-2A-MCS on the hPGK promoter, linker and PuroR. These two groups of vectors are all lentiviral (LV) or retroviral (RV) vectors, which lack poly(A) signals in the expression cassettes of foreign GOIs 24, 25, therefore are highly vulnerable to aberrant splicing when foreign GOI is inserted for expression. The p3×Flag-CMV-10-like vector has the same gene organization as p3×Flag-CMV-10. The SV40-Neo/KanR cassette is directly located downstream of the hGH poly(A) of the foreign GOI. The hGH poly(A) fails to terminate the transcript of GOIs efficiently, and the readthrough transcript is liable to be aberrantly spliced.

## Discussion

Comparison of the expression patterns of recombinant hFACI and mFACI proteins in cultured cells revealed an extra 37-kD protein band when hFACI was expressed from three expression constructs p3xFlag-CMV-hFACI, pLVX-V5-hFACI and pCW57-V5-hFACI. The extra band was the product of aberrant splicing between hFACI ORF and downstream plasmid sequence. Whereas the splice donor sites located at the EJ of hFACI ORF remained unchanged, the splice acceptor sites varied among the three constructs and were more difficult to predict. It will therefore be of interest to determine whether the aberrant splicing might be driven by the splice donor sequence in the GOI. In other words, once a functional splice donor site is available on the sequence of the foreign GOI, the aberrant splicing would occur as long as a splice acceptor-like sequence is found on the downstream vector sequence.

Human *FACI* is not the only gene with the risk for vector-mediated aberrant splicing. In this study, we performed whole genome analysis and identified most, but not all, human and mouse genes susceptible to vector-dependent aberrant splicing. At this first stage, our analysis is limited to protein-coding genes. We will continue to analyze non-coding genes in future since aberrant splicing on the EJ of non-coding genes should also exist 26, 27. Genes whose EJ sequences match non-canonical splicing donors were not discussed here. Genes prone to aberrant splicing on cryptic splice sites were identified but not systematically analyzed in this study.

Readthrough transcripts are commonly degraded by the nuclear RNA exosome 28. Our study showed that most aberrantly spliced mRNAs derived from readthrough transcripts are successfully translated but not degraded, suggesting that the action of nuclear RNA exosome on readthrough transcripts derived from exogenously introduced expression plasmids might not be complete. The aberrant fusion protein generated could severely affect the function of the WT protein. Notably, the aberrant proteins could be more abundant than WT proteins. In our study, the 37-kD extra band was more pronounced than that of WT hFACI in most cases. One possible reason is that splicing might increase translational efficiency 29. The fusion protein translated from aberrantly spliced transcript shares a portion of amino acid sequence with the WT protein, hence it might serve as a dominant-active or dominant-negative form 30, perturbing the function of the WT protein.

V5 epitope tag is a widely used peptide tag in biomedical studies 31. In our study, all 17 human and mouse genes tested underwent aberrant splicing between C-terminal V5 tag and downstream vector sequence, indicating that such aberrant splicing frequently occurs in most if not all genes susceptible to vector-mediated aberrant splicing. Furthermore, aberrant splicing on V5 tag should not just occur in the selected genes. Essentially all genes, which recruit spliceosomes, could be vulnerable to V5 tag-mediated aberrant splicing. Aberrant splicing between the V5 tag and downstream sequence generates an aberrant protein, which does not react to anti-V5 antibody but could impair the function of the WT protein. A previous study has reported aberrant splicing on V5 tag in V5-tagged DUX4 transgenic mice 32. Our demonstration of the frequent vector-mediated aberrant splicing on V5 tag is noteworthy. Apart from V5 tag, EGFP and some other widely used protein tags could also provide cryptic splicing sites, leading to vector-mediated aberrant splicing during gene expression, as shown in another study 33. Thus, there is an urgent need for recoding the currently used protein tags as exemplified in our work.

To minimize the impact of vector-mediated aberrant splicing effects on GOI expression, several strategies can be considered. First, recoding splice sites is the most effective way to reduce or eliminate aberrant splicing. However, several technical difficulties could be encountered. For example, the donor sites in the GOIs have to be identified and experimentally validated, which could be time-consuming and laborious. In some cases, synonymous mutation is impossible, because disruption of the splice sites sometimes results in the changes of protein sequence. Moreover, codon usage bias may affect translation efficiencies or even render translation unsuccessful 34. Second, strong transcriptional terminators should be included in the expression cassette of GOI. Terminators stop the transcription to avoid production of redundant pre-mRNA. Strong terminators are particularly recommended, since weak terminators seem to be difficult to terminate all readthrough transcription 35, 36. Third, for lentiviral and retroviral vectors, expression cassettes of transgenes are commonly cloned without polyadenylation signals 25. To minimize the impact of aberrant splicing on lentiviral and retroviral vectors, it is better to make downstream sequences of GOIs as short as possible. For instance, expression cassettes of selectable markers might be placed upstream of the expression cassettes of foreign GOIs. Finally, vector sequence can be optimized. For example, putative or empirically identified splice acceptor sites should be recoded to avoid aberrant splicing. In this regard, it is known that sequence information in addition to the consensus is required in the selection of splice sites 37.

Our study has revealed that a subset of human and mouse genes are particularly susceptible to vector-mediated aberrant splicing if inserted into certain expression vectors. Production of the resulting fusion proteins could substantially undermine the accuracy of gain-of-function studies and, more importantly, cause unexpected side effects in transgene-based gene therapy. Previous studies on aberrant splicing in relation to gene therapy focus primarily on splicing events between the therapeutic transgene and cellular genome after lentiviral integration. Particularly, in a clinical trial for gene therapy of β-thalassemia, an aberrantly spliced mRNA encoding a truncated HMGA2 protein has acquired clonal dominance and exhibited a beneficial effect 38. A similar event caused by lentiviral transduction of hematopoietic stem and progenitor cells in rhesus macaques has also resulted in clonal hematopoiesis ascribed in part to aberrant splicing of *PLAG1* encoding a transcription factor 39. A more systematic analysis of this type of vector-induced aberrant splicing by transcriptomics and semi-quantitative PCR has revealed a surprising abundance of readthrough transcription and aberrant splicing, which gives rise to lentiviral-cellular fusion transcripts 40, 41. Our report of another type of vector-mediated aberrant splicing involving the therapeutic transgene and downstream vector sequence provides another example in which aberrant splicing might also affect the therapeutic effects. Although we demonstrated it in cultured cells transiently and stably expressing foreign GOIs via expression vectors, this type of aberrant splicing should also occur when GOI-expressing cells are introduced into humans and mice *ex vivo*. Scenarios where aberrant splicing might occur include gene therapy based on transgene expression 42, derivation of induced pluripotent stem cells through lentiviral expression of Yamanaki factors 43, 44, generation of CAR-T cells engineered with lentiviral vectors 45, 46, and expression of immunogenic antigens from DNA vaccines on plasmid constructs 46. In many cases, transgenic-lentiviral fusion transcripts are also formed in addition to the cellular-lentiviral fusion transcripts. Plausibly, more complicated aberrant splicing involving the transgene, the lentiviral vector and the cellular genome should also occur, leading to altered biological effects that require attention and further investigations.

## Materials and methods

### Materials and reagents

Reagents, plasmids, primers, and antibodies have been listed in Supplementary Table S1. ORFs of human *FACI* and mouse *Faci* were subcloned into pLVX-Puro, pCW57-GFP-2A-MCS, pLVX-mCherry-C1 and p3×Flag-CMV-10 vectors. Constructs pLVX-hFACI-add93H, pLVX-hFACI-R94K, pLVX-hFACI-V93I and pLVX-hFACI-QVR were derived from pLVX-V5-hFACI by site-directed mutagenesis. Other plasmids were constructed by us or by Synbio Technologies (Suzhou, China) and BGI Technologies (Shenzhen, China).

### Site-directed mutagenesis

pLVX-V5-hFACI was mutated by Q5 Site-Directed Mutagenesis Kit (New England Biolabs). Mutation primers were designed by online software NEBaseChanger. PCR, kinase-ligase-DpnI (KLD) treatment and transformation were conducted following the manufacturer's instructions. All mutations had been confirmed by DNA sequencing (BGI).

### Cell culture and transfection

Human embryonic kidney cell line HEK293T was cultured in Dulbecco's Modified Eagle's Medium (DMEM, ATCC) containing 10% fetal bovine serum (FBS, Life Technologies). Mouse immortal hepatic cell line AML12 was cultured in DMEM/F-12 medium (Gibco) supplemented with insulin, transferrin, selenium (ITS; Gibco), 40 ng/mL dexamethasone (Sigma) and 10% FBS. All cell lines were maintained at 37°C with a humidified atmosphere containing 5% CO_2_.

HEK293T cells were transfected via GeneJuice transfection reagent (Novagen). AML12 cells were transfected using Lipofectamine 3000 (Invitrogen) and P3000 (Invitrogen). Transfection procedures were performed as instructed.

### Stable cell line generation

Stable cell lines were generated with a lentiviral vector system as previously described 47. Briefly, the lentiviral backbone plasmid was co-transfected into HEK293T cells with packaging plasmids. After 48-hour incubation, the culture medium was collected and filtered through a 0.22-µm filter. Virus-containing supernatant was further concentrated with Lenti-X™ Concentrator (Takara) and added to pre-seeded target cells with 8 μg/ml polybrene. The transduction was allowed to proceed for 48 hours, followed by puromycin selection to kill the lentivirus-negative cells.

### Protein extraction and immunoblotting

Immunoblotting was performed as previously described 17, 48. Cell or tissue samples were lysed in RIPA lysis buffer (25 mM Tris-HCl, pH 7.4, 150 mM NaCl, 1% NP-40, 0.1% sodium dodecyl sulfate, 0.5% sodium deoxycholate) supplemented with protease inhibitor cocktails (Roche) by incubation for 30 min at 4°C. The protein concentration of lysates was measured using the Bradford dye-binding method (BioRad). Protein samples were separated by SDS-PAGE, electroblotted onto polyvinylidene difluoride membranes (Millipore), incubated with primary and secondary antibodies sequentially and visualized by enhanced chemiluminescence (Amersham). Experiments were performed with three independent biological replicates.

### Immunoprecipitation, Coomassie blue staining, and mass spectrometry

For immunoprecipitation, cell lysates were incubated with anti-V5 antibody and Dynabeads at 4 ^o^C overnight as previously described 17, 49. After incubation, the beads were washed three times with the wash buffer and the precipitates were boiled with the 2× protein loading buffer. Around one-tenth of the samples were separated by SDS-PAGE followed by immunoblot analysis. The remaining eluted samples were separated by SDS-PAGE and visualized by Coomassie blue staining. The 37-kD band was collected and sent to the Centre for PanorOmic Sciences (CPOS) of the University of Hong Kong for mass spectrometry. The mass spectrometric data were analyzed by proteomics software MaxQuant.

### RNA extraction and RT-PCR

Total RNA was extracted with RNAiso Plus reagent (TaKaRa) according to the manufacturer's instructions. Extracted RNA was incubated with DNase I (Ambion) at 37 ℃ for 30 min to digest the remaining genomic DNA. Reverse transcription was performed using Transcriptor First Strand cDNA Synthesis reagents (Roche). PCR was then conducted using KAPA HiFi HotStart ReadyMix (Kapa Biosystems). Experiments were performed with three independent biological replicates.

### Molecular cloning and sequencing

The PCR fragments from RT-PCR were purified by QIAquick PCR & Gel Cleanup Kit (QIAGEN). The purified PCR products were then cloned into the pGEM^®^-T Vector System (Promega) by TA cloning. Ligation, transformation and blue-white screening were all carried out as per manufacturers' instruction. The positive clones were selected and sent to BGI Technologies for DNA sequencing.

### Whole genome analysis of genes susceptible to vector-dependent aberrant splicing

All gene sequences and their annotations were retrieved from the Ensembl website (www.ensembl.org). Only human and mouse protein-encoding genes registered in the UniProtKB/Swiss-Prot database were selected for analysis. DNA sequence cognate to mRNA was analyzed. First, genes with at least one EJ matching “N|GT” sequence were retrieved, which formed a list of genes prone to vector-dependent aberrant splicing. Furthermore, genes whose EJs match “N|GT” but are not located in the protein-coding region were excluded. After the above steps, refinement of the gene lists was completed (Supplementary Tables S2 and S3). Among these genes, a subset of genes containing at least one EJ matching “N|GTpuAG” was further selected to form the lists of genes that satisfy more stringent criteria (Supplementary Tables S4 and S5).

A total of 17 genes were selected from Supplementary Tables S4 and S5 for experimental validation. The 17 genes, together with their annotations and ORF sequence, were listed in Supplementary Table S6.

## Supplementary Material

Supplementary figures.

Supplementary tables.
