# Supplementary material for: Aberrant splicing events caused by insertion of genes of interest into expression vectors
Source: Int J Biol Sci. 2022 Jul 18;18(13):4914–31. doi: 10.7150/ijbs.72408 (PMC9379414; doi:10.7150/ijbs.72408)
Supplement: Supplementary file 1 — Supplementary figures. [file ijbsv18p4914s1.pdf]

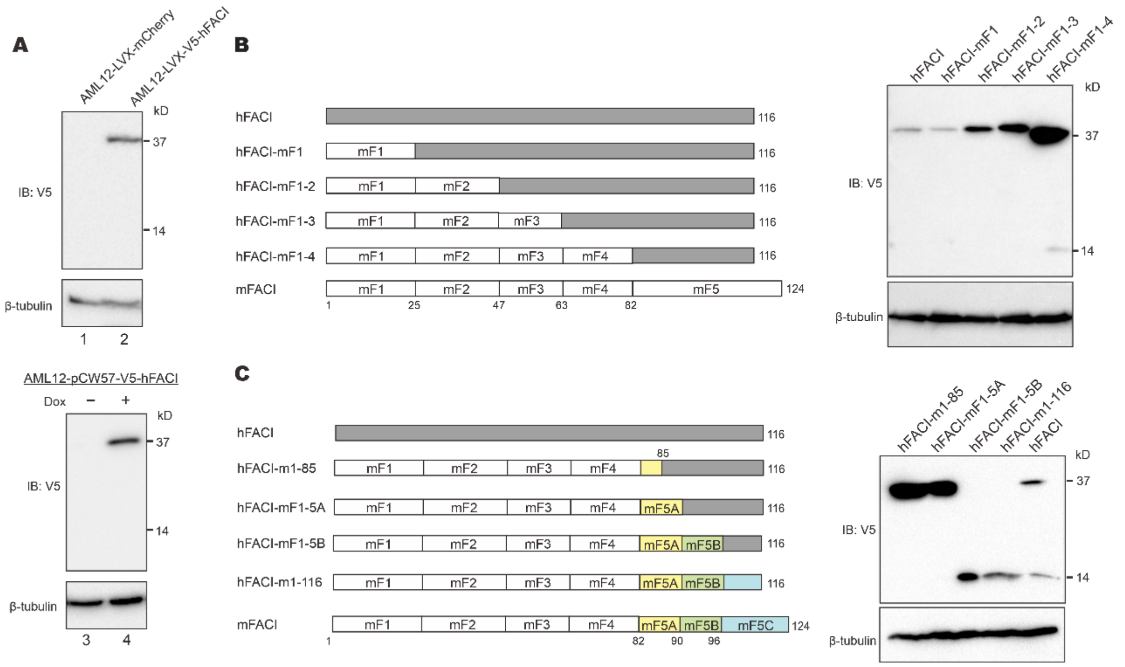

Figure S1. Expression of large-sized hFACI proteins from different constructs. (**A**) Immunoblotting. AML12 lines stably expressing V5-hFACI and mCherry were generated using pLVX-V5-hFACI and pLVX-mCherry-C1 (lanes 1-2). The AML12 stable cell line for inducible expression of V5-hFACI was generated based on pCW57-V5-hFACI (lanes 3-4). V5-hFACI expression in the three cell lines was detected by immunoblotting with anti-V5. Dox: doxycycline. (**B**) A list of human-mouse chimeric FACI constructs (left) and their protein expression (right). All chimeric constructs were cloned into the pLVX-Puro vectors with V5-tag. (**C**) A list of human-mouse chimeric FACI constructs were generated using pLVX-V5-mF1-4 (left). Protein expression from the chimeric constructs was detected by immunoblotting (right).

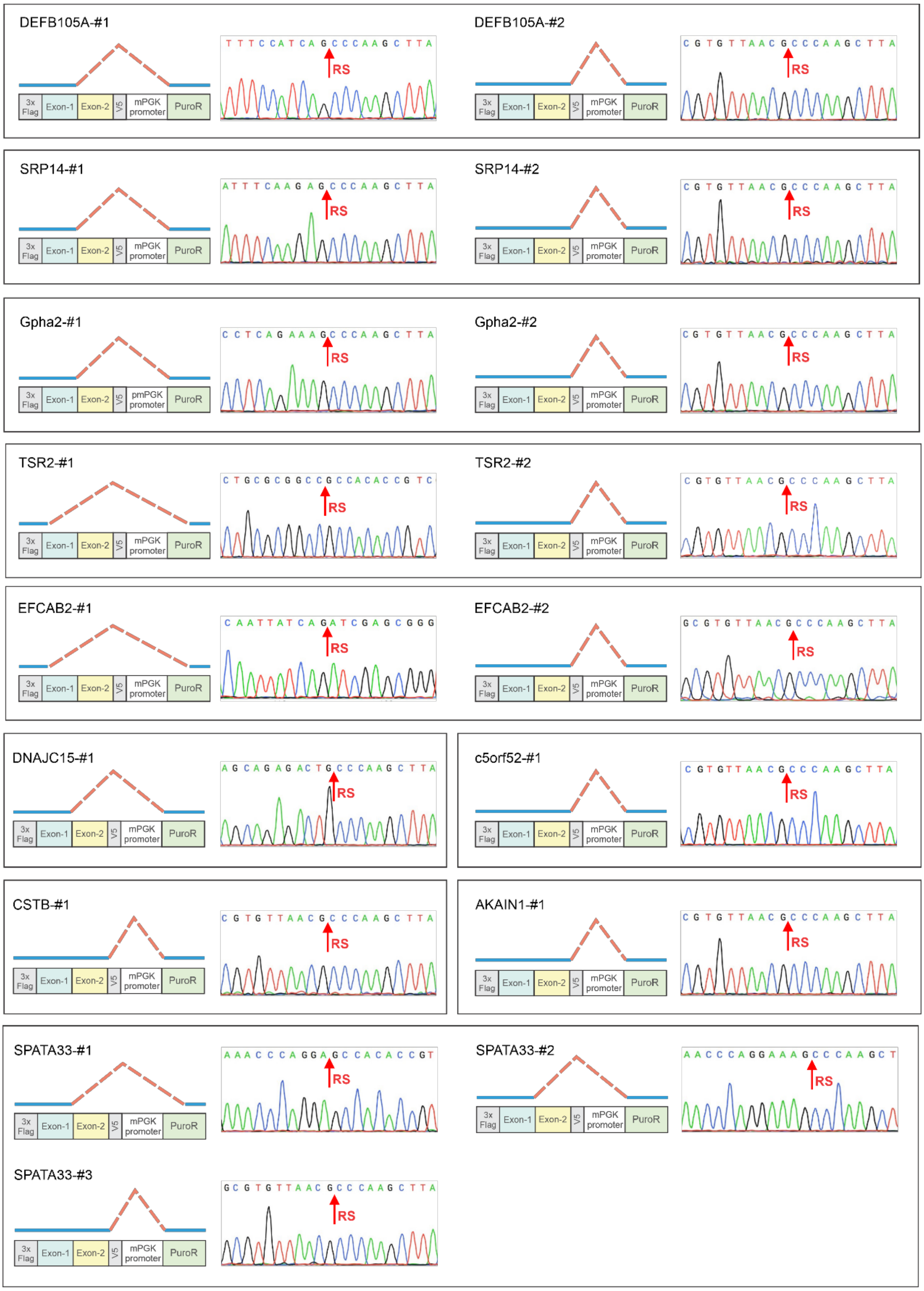

Figure S2. Further analysis of aberrant splicing in the indicated GOIs. Schematic diagrams and sequencing chromatograms of aberrantly spliced transcripts of 10 out of the 17 genes selected in Figure 3A. RS: the site of splice junction.

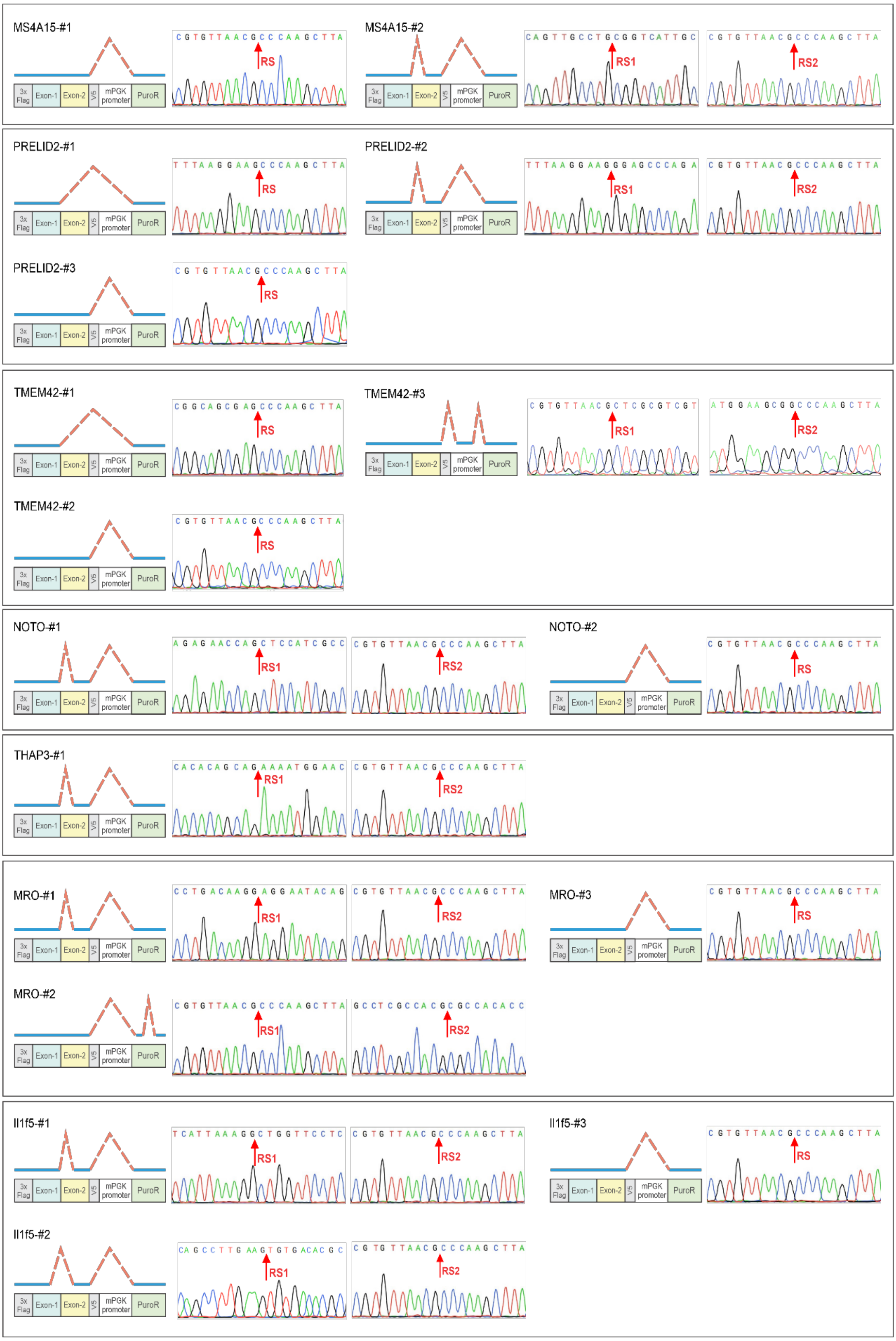

Figure S3. Further analysis of aberrant splicing in the indicated GOIs. Schematic diagrams and sequencing chromatograms of aberrantly spliced transcripts of the remaining 7 genes out of the 17 genes selected in Figure 3A. RS: the site of splice junction.

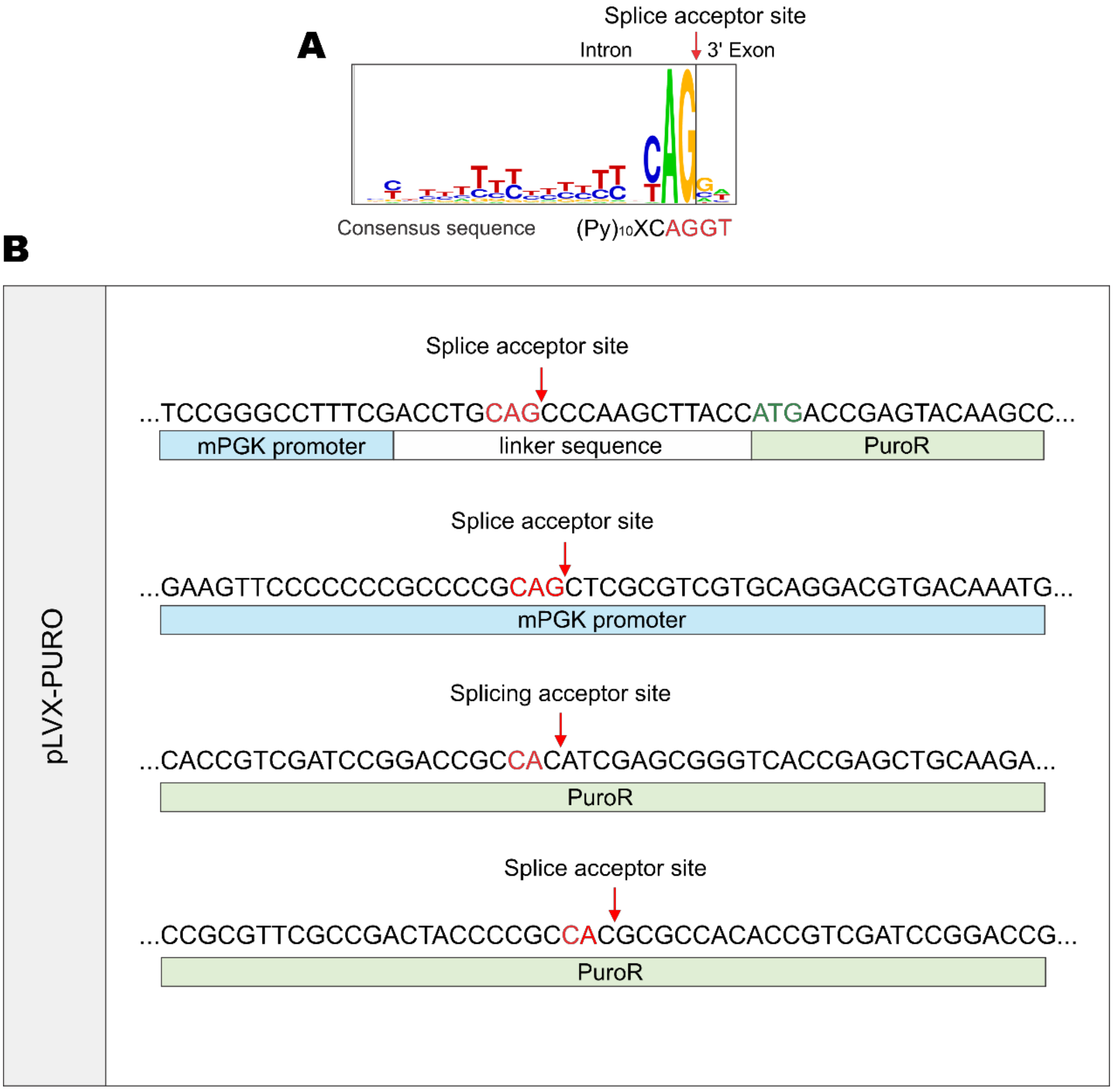

Figure S4. Splice acceptor sites in pLVX-Puro. (**A**) Consensus sequence of the splice acceptor site [4, 27]. (**B**) Four splice acceptor sites were identified on the pLVX-Puro vector by RT-PCR and sequencing. The splice acceptor site within the linker sequence between the mPGK promoter and PuroR mediates most aberrant splicing events. Rarely, two sequences in PuroR and a sequence in mPGK promoter could also function as splice acceptor sites.

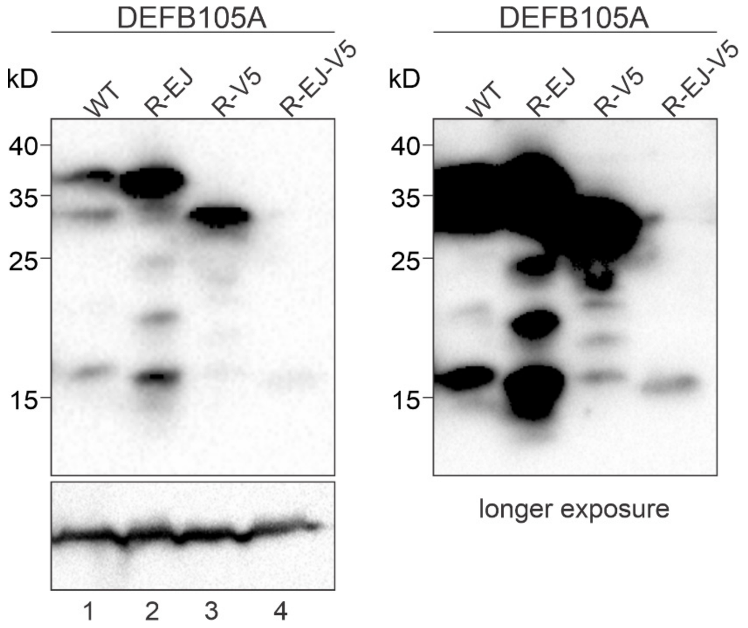

Figure S5. Western blot analysis of DEFB105A. A longer exposure immunoblot image of DEFB105A in Figure 5D is presented to reveal faint bands.
